# Contribution to the genus *Filipinolotis* Miyatake, 1994 (Coleoptera, Coccinellidae, Sticholotidini)

**DOI:** 10.3897/zookeys.793.24790

**Published:** 2018-10-29

**Authors:** Yanqing Lv, Xiaoning Zhang, Adam Ślipiński, Yurong He, Xingmin Wang

**Affiliations:** 1 Key Laboratory of Bio-Pesticide Innovation and Application, Engineering Technology Research Center of Agricultural Pest Biocontrol, Guangdong Province; Department of Entomology, South China Agricultural University, Guangzhou 510640, China South China Agricultural University Guangzhou China; 2 Zengcheng Entry-exit Inspection and Quarantine Bureau, Guangzhou 511340, China Zengcheng Entry-exit Inspection and Quarantine Bureau Guangzhou China; 3 CSIRO Ecosystem Sciences, Australian National Insect Collection, GPO Box 1700, Canberra, ACT 2601, Australia CSIRO Ecosystem Sciences, Australian National Insect Collection Canberra Australia

**Keywords:** Coccinelloidea, Coleoptera, *
Filipinolotis
*, Luzon, new species, Philippines, Sticholotidini

## Abstract

The genus *Filipinolotis* Miyatake has been reviewed in this study. Descriptions and illustrations of two species (*F.latefasciata* Miyatake and *F.purpuratorotunda* Wang, Zhang & Ślipiński, **sp. n.**) in the Luzon island of the Philippines, are given. The male genitalia of *F.latefasciata* are described for the first time. A key to known species is also provided.

## Introduction

The family Coccnellidae is an abundant species group of beetles. Ślipiński (2007) proposed a two subfamily system with Microweiseinae and Coccinellinae, merging Sticholotidini into an expanded concept of Coccinellinae (Escalona and Ślipiński 2012). The tribe Sticholotidini was defined by Gordon (1977) and Miyatake (1994). The final composition of Sticholotidini and the taxonomic status of many genera are not fully resolved (Ślipiński 2004). Coccinellid evolutionary history is borne out in previous molecular studies (Giorgi et al. 2009; Seago et al. 2011; Robertson et al. 2015).

Miyatake (1994) revised the Asian genera of the tribe Sticholotidini and proposed six new genera: *Synonychimorpha*, *Chilocorellus*, *Sulcolotis*, *Filipinolotis*, *Mimoserangium*, and *Coelolotis* for the species described from China, Philippines, and Vietnam. The genus *Filipinolotis* was erected with *F.latefasciata* Miyatake, 1994 as the type species from Luzon Island, Philippines. *Filipinolotis* is similar to *Sticholotis* except for some differences in characters of the hind wings (atrophied), antennae (shorter with distinct club), prosternal process (raised), and epipleura (broad). To date, *F.latefasciata* has been the only species in the genus *Filipinolotis*. In this paper, *F.purpuratorotunda*, sp. n. from Luzon Island in the Philippines is described and added to this genus.

## Materials and methods

The specimens examined were collected from Luzon, Philippines. All materials were deposited at Australian National Insect Collection, CSIRO, Canberra, Australia (**ANIC**), South China Agricultural University, Guangzhou, China (**SCAU**), and Museum für Naturkunde in Berlin, Germany (**MNB**). External morphology was observed with a dissecting stereoscope (Zeiss Stemi 2000-cs). The following measurements were made with an ocular micrometer:

**TL** total length, length from apical margin of clypeus to apex of elytra;

**TW=EW** total width, width across both elytra at widest part;

**TH** height, from the highest part of the beetle to elytral outer margins;

**HW** head width in a frontal view, widest part including eyes;

**PL** pronotal length, from the middle of anterior margin to the base of pronotum;

**PW** pronotal width at widest part;

**EL** elytral length, along the suture, from the apex to the base including the scutellum.

Images were taken with digital cameras (AxioCam HRc and Coolsnap-Procf & CRI Micro*Color) connected to a dissecting microscope in the Key Laboratory of Bio-Pesticide Innovation and Application, Engineering Technology Research Center of Agricultural Pest Biocontrol, Guangdong Province of South China. The software AxioVision Rel. 4.8 and Image-Pro Plus 5.1 were used to capture images from the cameras. Software Adobe Photoshop CC 2015 was used for cleaning up images.

Terminology follows Ślipiński (2007) and Ślipiński and Tomaszewska (2010). Type specimens designated in the present paper are deposited at ANIC and SCAU.

## Taxonomy

### Filipinolotis

Taxon classificationAnimaliaColeopteraCoccinellidae

Miyatake, 1994

Filipinolotis Miyatake, 1994: 254. Type species: Filipinolotislatefasciata Miyatake, 1994.

#### Diagnosis.

**Figure 1.**
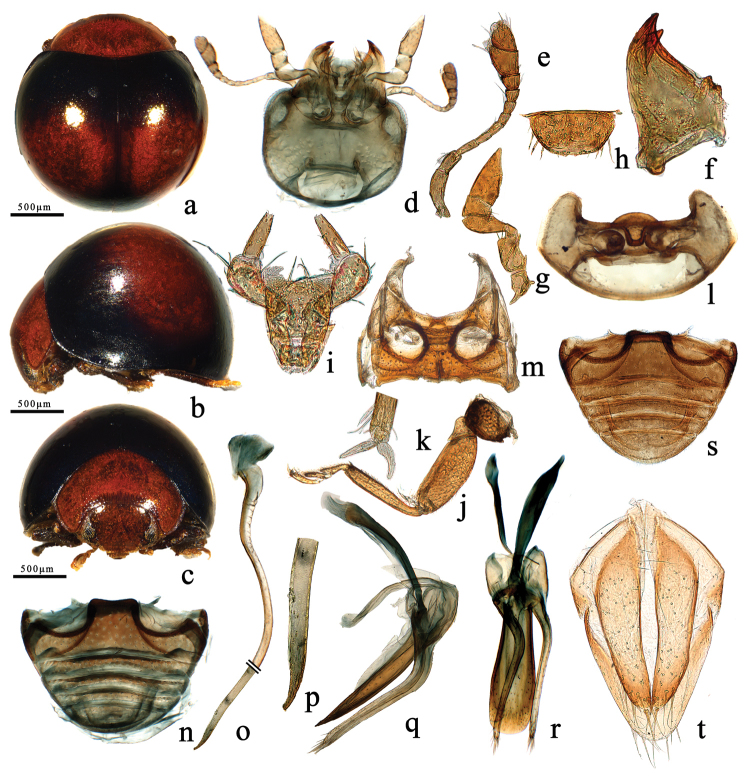
Morphological characters of the genus *Filipinolotis*. **a–t***F.latefasciata* Miyatake. **a** dorsal habitus **b** lateral habitus **c** frontal habitus **d** head, ventral **e** antenna **f** mandible **g** maxilla **h** labrum **i** labium **j** front leg **k** tarsal claws **l** prothorax, ventral **m** mesoventrite and metaventrite **n** male abdomen **o** penis **p** apex of penis **q** tegmen, lateral view **r** tegmen, ventral view **s** female abdomen **t** ovipositor.

**Figure 2.**
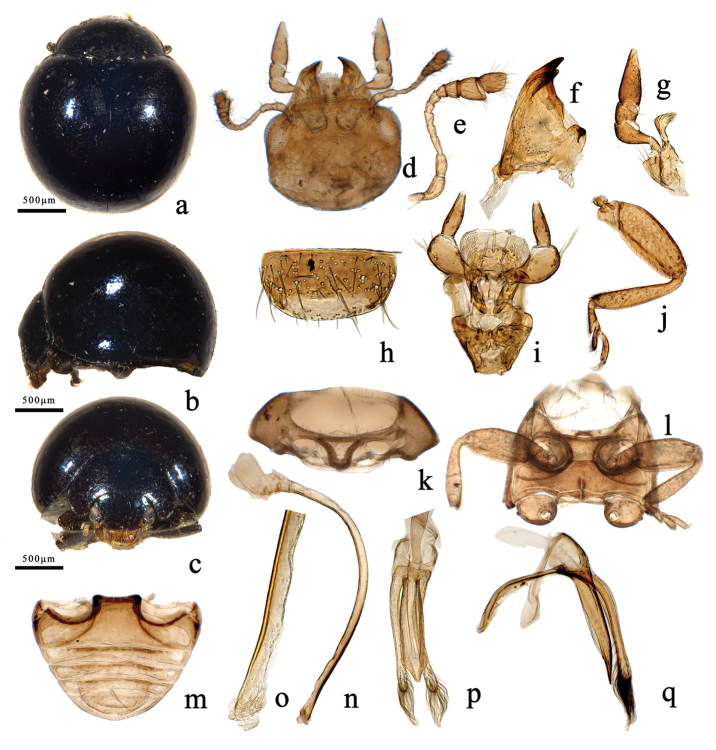
*Filipinolotispurpuratorotunda* sp. n. **a** dorsal habitus **b** lateral habitus **c** frontal habitus **d** head, ventral **e** antenna **f** mandible **g** maxilla **h** labrum **i** labium **j** front leg **k** prothorax, ventral **l** mesoventrite and metaventrite **m** abdomen **n** penis **o** apex of penis **p** tegmen, ventral view **q** tegmen, lateral view.

The genus *Filipinolotis* can be distinguished from other genera of the tribe Sticholotidini by the following characters: body rounded, glabrous, dorsum strongly convex (Figs 1a–c, 2a–c); antennae with 11 antennomeres (Figs 1e, 2e); hind wings atrophied; elytral epipleuron unevenly broadened; abdomen with five ventrites, abdominal postcoxal lines incomplete (Figs 1n, s, 2m).

#### Description.

*Body* rounded, dorsum strongly convex and glabrous (Figs 1a–c, 2a–c). Head smooth, with sparse short hairs (Figs 1c, 2c). Antennae with 11 antennomeres, scape and pedicel robust, pedicel shorter than scape; flagellum 9-segmented, gradually broadening towards apex, last-three antennomeres forming a fusiform club (Figs 1d–e, 2d–e).

*Clypeus* protruded with anterior margin slightly emarginate at middle (Figs 1c, 2c). Labrum transverse, narrowly rounded and covered with dense, long setae (Figs 1h, 2h). Mandible with two apical teeth, sharp and smooth without additional denticles (Figs 1d, f, 2d, f). Maxillary palp 4-segmented with terminal segment cut obliquely at apex (Figs 1d, g, 2d, g). Labial palpomere 3-segmented, with robust 2^nd^segment and slender terminal segment, ligula membranous and plump, insertion of labial palps is anterior on the prementum (Figs 1i, 2i).

*Pronotum* transverse, strongly convex, emarginate at anterior margin, hind margin bent into a curved shape; lateral margin slightly oblique, with fine groove continuing towards basal corner from anterior corner, anterior angles rounded and smooth, hind angles obtuse and smooth (Figs 1a–c, 2 a–c). Scutellum minute, triangular (Figs 1a, c; 2a, c). Elytra without humeral angles, strongly convex, almost as wide at anterior margin as hind margin of pronotum, lateral margins extremely narrow; lateral margins with narrow rim, invisible from above (Figs 1a–c, 2a–c). Hind wings atrophied.

*Prosternum* T-shaped. Prosternal process narrowed, less than 0.6 times width of coxal diameter and not prominent anteriorly between coxae, prosternal carinae present and fan-shaped (Figs 1l, 2k). Mesoventrite trapezoid-shaped, coarsely keeled on the anterior edge, metaventrite as long as mesoventrite (Figs 1m, 2l). Elytral epipleuron wide and uneven, gradually narrowed to apex. Legs with pubescence rather dense, coxae square-shaped, obvious robust; trochanter triangular, robust; femora thick, deeply grooved beneath for tibiae when retracted; length of tibia subequal to femur, slender, and widening but not angulate outwardly; tarsi 4-segmented; claws simple and slender, without teeth (Figs 1j, k, 2j). Abdomen with five ventrites in both sexes, ventrite 1 length measured below coxal cavity, almost as long as ventrites 2^nd^ to 4^th^ combined, abdominal postcoxal lines incomplete, not recurved but meeting hind margin of ventrite 1 (Figs 1n, s, 2m).

*Male genitalia*: tegmen stout, basal piece with distinct strut and additional, dorsal strut-like projection, parameres well developed and setose apically. Penis uniformly tubular, curved (Figs 1o–r, 2n–q).

*Female genitalia*: coxites long triangular with interior margin slightly emarginate, setose apically; styli conspicuous (Figure 1t).

#### Distribution.

Philippines.

##### Key to the species of *Filipinolotis*

**Table:** 

1	Pronotum reddish yellow, elytra blackish and shiny, with a broad transverse reddish yellow band in middle (Figure 1a–c); apex of penis gradually narrowing down (Figure 1o, p); penis guide of tegmen in ventral view broad and flat, rounded with uniform setae apically (Figure 1q, r)	***F.latefasciata* Miyatake**
–	Pronotum and elytra purplish to black, shiny, without any spots (Figure 2a–c); apex of penis tubular with membranous gonopore at apex (Figure 2n, o); penis guide of tegmen in ventral view narrow and symmetrical, horn-shaped apically (Figure 2p, q)	***F.purpuratorotunda* Wang, Zhang & Ślipiński, sp. n.**

### Filipinolotis latefasciata

Taxon classificationAnimaliaColeopteraCoccinellidae

Miyatake, 1994

Figure 1

Filipinolotis latefasciata Miyatake, 1994: 255

#### Diagnosis.

This species can be recognized by the following combination of characters: pronotum reddish yellow, elytra blackish shiny with a broad transverse reddish yellow band in the middle (Figure 1a–c); penis guide of tegmen in ventral view broad and flat, with a rounded apex, parameres narrow and almost straight with dense setae at apices (Figure 1q–r); penis uniformly tubular, curved, gradually narrowing to apex (Figure 1o–r).

#### Description.

TL: 1.92–2.3 mm, TW: 1.86 mm, TH: 1.35–1.38 mm, TL/TW: 1.03–1.24; PL/PW: 0.38–0.42; EL/EW: 0.89–1.08; HW/TW: 0.39; PW/TW:0.67–0.74.

Head reddish yellow (Figure 1c, d). Pronotum reddish yellow (Figure 1a–c). Scutellum blackish shiny (Figure 1a, c). Elytra blackish and shiny, with a broad transverse reddish yellow band at middle, covering almost half of elytra (Figure 1a–c). Underside blackish brown, except claws yellowish.

Body rounded, dorsum strongly convex and glabrous (Figure 1a–c). Head large, with sparse short golden pubescence (Figure 1d). Pronotum glabrous with uniformly minute and dense punctures (Figure 1a–c). Elytra convex, entire surface uniformly punctate (Figure 1a–c). Elytral epipleuron with sparse golden pubescence. Prosternum with sparse golden pubescence and inconspicuous punctures (Figure 1l). Mesoventrite and metaventrite with sparse golden pubescence and inconspicuous punctures, mesoventrite with narrow intercoxal process, chin piece formed on the anterior margin of mesoventrite; metaventrite short, as wide as mesoventrite (Figure 1m). Legs with dense golden pubescence (Figure 1j, k). Abdomen with sparse golden hairs and uniform punctures (Figure 1n, s).

Male genitalia (Figure 1o–r). Tegmen stout and symmetrical, penis guide in lateral view wide at base and gradually narrowing to pointed apex, the basal 2/3 with membranous prominence; in ventral view broad and flat, rounded apically; parameres narrow and almost straight with dense setae at apices (Figure 1q–r), phallobase membranous. Penis simple tubular, curved, gradually narrowing along apical third to pointed apex, penis capsule membranous, without inner arm (Figure 1o–p).

#### Material examined.

Philippines: 1 male, Mt. Polis, Lepanto, Luzon Prov., Böttcherleg (MNB); 1 female, Mt. Data, Mountain, Luzon Prov., 7500 ft, 23.iv.1946; 1 male Philippines, Luzon (ANIC).

#### Distribution.

Philippines (Luzon).

### Filipinolotis purpuratorotunda

Taxon classificationAnimaliaColeopteraCoccinellidae

Wang, Zhang & Ślipiński sp. n.

http://zoobank.org/D188809E-F91D-44D4-853E-BB53986EBCA6

Figure 2

#### Diagnosis.

This species is similar to *F.latefasciata* Miyatake but it can be distinguished from the latter as follows: body uniformly black with purple shine, without spots (Figure 2a–c); penis guide of tegmen in ventral view narrow with a nipple-shaped apex (Figure 2p, q); apex of the penis truncate, membranous (Figure 2o). In *F.latefasciata*, pronotum reddish yellow, elytra blackish and shiny, with a broad transverse reddish yellow band in the middle (Figure 1a–c); penis guide of tegmen in ventral view broad and flat, with rounded apex (Figure 1r); apex of the penis pointed (Figure 1o, p).

#### Description.

TL: 1.99–2.63 mm, TW: 1.84–2.55 mm, TH: 1.28–1.8 mm, TL/TW: 1.03–1.08; PL/PW: 0.40–0.49; EL/EW: 0.93; HW/TW: 0.34–0.39; PW/TW: 0.59–0.60.

*Color*: Head, pronotum, scutellum, elytra purplish shiny black, without spots (Figure 2a–c). Underside blackish brown, except yellowish claws.

*Body* rounded, dorsum strongly convex and glabrous (Figure 2a–c). Head large with scattered short golden pubescence (Figure 2c, d). Pronotum glabrous, with uniformly distributed punctures (Figure 2a–c). Scutellum of isosceles triangle shape (Figure 2a, c). Elytral surface uniformly punctate (Figure 2a–c). Prosternum, mesoventrite, and metaventrite with scattered golden pubescence and inconspicuous punctures (Figure 2k, l). Elytral epipleuron with uniform golden pubescence. Legs with dense golden pubescence (Figure 2j, l). Abdomen with sparse golden pubescence and uniformly distributed punctures (Figure 2m).

*Male genitalia*. Tegemen stout and symmetrical, tegminal strut stout and straight; penis guide in lateral view almost straight and gradually narrowing along apical third to pointed apex, with membranous prominence at basal half; in ventral view narrowest at base and gradually broadening to apex, then strongly narrowed to form a nipple-shaped apex; parameres with dense long setae apically, longer than penis guide; phallobase membranous (Figure 2p, q). Penis uniformly tubular, curved, basal capsule membranous, without distinct arms; apex of penis subtruncate with membranous appendage (Figure 2n, o).

#### Types.

Holotype: Philippines: 1 male, La Trinidad, Luzon Prov., 1300m, 4–5.IV.1968, Benqae (ANIC); Paratypes: Philippines: 1 male, Island of Basilan, Baker (ANIC); 1 male, Disimungal, Madela, Quirino, Eastern Luzon, XII.2014 (SCAU).

#### Disribution.

Philippines (Luzon).

#### Etymology.

The name *purpuratorotunda* is composed of the word *purpuratus*, which refers to the purplish color of the body and *rotundus*, referring to the rounded body shape.

## Supplementary Material

XML Treatment for Filipinolotis

XML Treatment for Filipinolotis latefasciata

XML Treatment for Filipinolotis purpuratorotunda
